# Clinicopathological Features and Outcomes of Granulosa Cell Tumor of the Ovaries - A Retrospective Study

**DOI:** 10.7759/cureus.38892

**Published:** 2023-05-11

**Authors:** Sameen Bin Naeem, Naqib U Baloch, Mussadique A Jhatial, Mansoor Abbas, Samir Fasih, Rizwan Masood Sheikh, Syed Abdul M Hamdani, Neelam Siddiqui

**Affiliations:** 1 Medical Oncology, Shaukat Khanum Memorial Cancer Hospital and Research Centre, Lahore, PAK

**Keywords:** chemotherapy, outcomes, surgery, prognostic factors, ovarian cancer, granulosa cell tumor

## Abstract

Background

Granulosa cell tumor (GCT) is rare among all ovarian cancers. Its overall prognosis is favorable; however, the presence of extra-ovarian disease is associated with worse clinical outcomes. We report a retrospective analysis of granulosa cell tumors to evaluate the clinicopathological features and their outcomes.

Methods

This retrospective study included 54 adult patients aged 13 years and older. After data extraction and scrutiny, only those patients who were treated and followed up later at our institute were included in this study.

Results

Fifty-four patients were evaluated in this study, with a median age of 38.5 years. Most of the patients had dysfunctional uterine bleeding and abdominal pain (40.7%, n=22). The majority (n=26, 48%) underwent completion surgery as per ovarian protocol; however, 16.7% (n=09) patients underwent simple total abdominal hysterectomy with a bilateral salpingo-oophorectomy (TAH+BSO), debulking surgery in 3.7% (n=2), unilateral salpingo-oophorectomy in 20.4% (n=11) and fertility-sparing surgery in 11.1% (n=06) of the patients. Pathological stage I-A was found in 59.3%(n=32), I-C in 25.9% (n=14), II-A in 1.9% (n=1), III-A in 1.9% (n=1), III-C in 9.3% (n=5) and IV-B in 1.9% (n=1) of the population. Eleven (20.3%) patients relapsed during their course of treatment. Out of these 11 patients, three went into remission, two still have active disease, and six patients died.

Conclusion

Post-menopausal patients, more advanced disease at presentation, capsular rupture, presence of ascites, omental involvement, peritoneal spread, and residual disease after surgical resection were the main contributing factors towards poorer outcomes affecting disease-free survival. Overall median disease-free survival was 60 months for all the stage groups, while the overall survival was 62 months.

## Introduction

Ovarian cancer is the most common gynecologic malignancy that results in cancer-related death worldwide. Epithelial ovarian malignancies, which mostly affect postmenopausal women, account for around 90% of tumors [1,2]. Ovarian sex cord-stromal tumors are rare, typically developing in the first two to three decades of life, accounting for around 7-10% of all primary malignant ovarian tumors. Adult granulosa cell tumors of the ovary are an exception, having a late onset, peak incidence between 50 and 55 years of age, accounting for only 2-5% of all ovarian cancers [3].

Based on histopathological features, ovarian granulosa cell tumors (GCT) can be classified as adult (95%) or juvenile (5%), have a fair prognosis, and have a propensity for late relapses. Surgery is the mainstay treatment option for primary and relapsed tumors [4]. Adjuvant chemotherapy is offered when high-risk features (stage 1-C, poor differentiation, high mitotic index, and tumor rupture) are identified in the surgical specimen or when the cancer is advanced at presentation, even though it has been fully resected [5]. The current preferred chemotherapy regimen is carboplatin plus paclitaxel doublet [6].

Overall, the prognosis for women with GCT is favorable; the five-year survival rate is more than 90%; however, the presence of extra ovarian disease is associated with a poorer five-year survival rate of 33-50% [7].

This retrospective analysis was carried out to evaluate the clinicopathological features and determine prognostic factors affecting the outcomes of granulosa cell tumors treated at our hospital, providing valuable data for researchers as no regional data is available in the literature.

## Materials and methods

Patients' data was taken from Shaukat Khanum Memorial Cancer Hospital and Research Center, Lahore Cancer registry department treated from January 1995 till December 2017, after obtaining institutional board review and ethical committee approval (EX-04-08-22-01). The data of 57 patients was obtained, and three patients were excluded based on no further treatment or follow-up. Because of the rarity of the disease, patients were included irrespective of age to include pediatric patients as well. Initial and post-treatment clinical features and treatment approaches, including age, performance status, presenting complaints, duration of symptoms, type of surgery, surgical and post-operative pathology findings, FIGO stage, chemotherapy given, residual disease post-surgery, radiological recurrence sites, post-recurrent treatment options were reviewed from the medical record. Data was further stratified for menopausal status, capsular rupture, presence of ascites, omental involvement, peritoneal spread, and residual disease after surgical resection.

Disease-free survival (DFS) and overall survival (OS) were analyzed by the Kaplan-Meier method. Further stratification of the data was carried out for post-menopausal status, advanced disease at presentation, capsular rupture, presence of ascites, omental involvement, peritoneal spread, and residual disease after surgical resection as effect modifiers for DFS and OS using the log-rank method. A p-value of less than 0.05 was considered a statistically significant difference.

Details obtained were tabulated and analyzed via Statistical Package for Social Sciences (SPSS) version 24 (IBM Inc., Armonk, New York).

Patient selection and treatment at our institute

Only diagnosed cases of granulosa cell tumors are present at our hospital. Staging of the disease is carried out, and further management is discussed in multidisciplinary team (MDT) meetings for further staging and treatment. Follow-up of the disease at the end of treatment is with periodic clinical examination, relevant blood tests, and scans as required. In case of recurrence, second-look surgery, chemotherapy, or palliative referral are advised after discussing the case in MDT.

## Results

This retrospective study included 54 patients aged 13 years and older, registered in our hospital from January 1995 till December 2017. The median age of our patient population was 38.5 years (range 13-77 years). Around 70% (n=38) of patients were premenopausal, and the remaining 30% (n=16) were postmenopausal. Eight patients (14.8%) were diagnosed with juvenile-type granulosa cell tumors of the ovary, the rest (85.2%) had adult-type granulosa cell tumors. Ten (18.5%) patients were nulliparous, while 44 (81.5%) were parous, with an average number of pregnancies of 2.98 (range 1-10). As far as clinical features are concerned, most of the patients presented with abnormal uterine bleeding (n=22, 40.7%) and abdominal pain (n=22, 40.7%), while abdominal distention (n=8, 14.8%) was the second most common presentation. The median duration of symptoms was five months.

**Figure 1:**
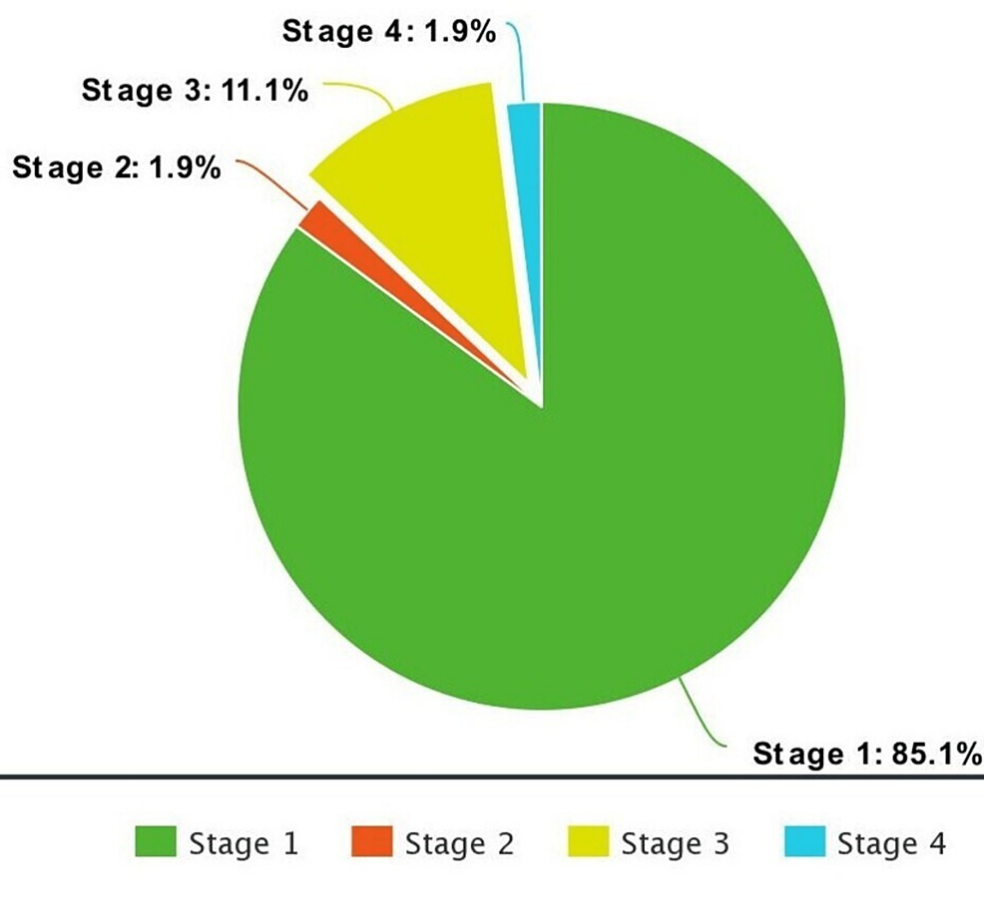
Pie chart showing FIGO stage percentages of the patients included in the study

Pre-operative imaging revealed right-sided lesions in 52% (n=28) of patients, left-sided lesions in 44% of patients (n=23), and only 4% had bilateral adnexal masses. The majority of patients (n=26, 48%) underwent completion surgery as per ovarian protocol; the rest of the patients (n=28, 52%) underwent simple total abdominal hysterectomy with bilateral salpingo-oophorectomy (TAH+BSO), debulking surgery, unilateral salpingo-oophorectomy and fertility-sparing surgery (FSS). Most of the patients (n=35, 64.8%) had intact ovarian capsules post-surgery, and the rest (n=19, 35.2%) had ruptured ovarian capsules. Peritoneal involvement was present in 13% (n=7), omental involvement in 13% (n=7), and ascites in 7.4% (n=4) of the patients. Pathological stage 1-A was found in 59.3% (n=32), I-C in 25.9% (n=14), II-A in 1.9% (n=1), III-A in 1.9% (n=1), III-C in 9.3% (n=5) and IV-B in 1.9% (n=1) of the population (Figure 1).

Post-operative imaging findings were consistent with no residual disease in 88.9% (n=48), while 11.1% (n=6) had residual disease. Adjuvant chemotherapy was given to 38.60% (n=22) of the patients (Table 1).

**Table 1:** Baseline patient and tumor characteristics

Characteristics		Number of patients	Percentage
Eastern Cooperative Oncology Group performance status (ECOG)	0	47	87%
	1	6	11.10%
	3	1	1.90%
Menstrual status	Postmenopausal	16	29.60%
	Premenopausal	38	70.40%
Parity	Nulliparous	10	18.50%
	Uniparous	6	11.10%
	Multiparous	38	70.40%
Presenting complaints	Abdominal pain	22	40.70%
	Dysfunctional uterine bleeding	22	40.70%
	Abdominal distension	8	14.80%
	Amenorrhea	2	3.70%
Duration of symptoms	Less than 3 months	16	29.60%
	3-6 months	21	38.90%
	More than 6 months	17	31.50%
Pre-operative imaging findings	Right adnexal mass	19	35.20%
	Left Ovarian cyst	13	24.10%
	Left adnexal mass	11	20.40%
	Right ovarian cyst	9	16.70%
	Bilateral adnexal masses	2	3.70%
Type of surgery	Completion surgery	26	48.10%
	Total abdominal hysterectomy + bilateral salpingo-oophorectomy	9	16.70%
	Fertility sparing surgery	6	11.10%
	Left salpingo-oophorectomy	6	11.10%
	Right salpingo-oophorectomy	3	5.60%
	Debulking surgery	2	3.70%
	Total abdominal hysterectomy + left salpingo-oophorectomy	1	1.90%
	Bilateral salpingo-oophorectomy	1	1.90%
Laterality	Left	24	44.40%
	Right	19	35.20%
	Bilateral	11	20.40%
Capsule rupture	Intact	35	64.80%
	Ruptured	19	35.20%
Ascites	No	50	92.60%
	Yes	4	7.40%
Peritoneal spread	Free of tumor	47	87.00%
	Positive	7	13.00%
Omental involvement	Free of tumor	47	87.00%
	Positive	7	13.00%
Tumor size	<10cm	27	50.00%
	>10cm	27	50.00%
Residual disease	No	48	88.90%
	Yes	6	11.10%
FIGO stage	I	46	85.20%
	II	1	1.90%
	III	6	11.10%
	IV	1	1.90%
Type of granulosa cell tumor	Adult	46	85.20%
	Juvenile	8	14.80%
Chemotherapy given	Yes	21	38.90%
	No	33	61.10%
Chemotherapy regimen	Bleomycin/ etoposide/ cisplatin	8	14.80%
	Carboplatin/ cyclophosphamide	1	1.90%
	Carboplatin/ etoposide	3	5.60%
	Carboplatin/ paclitaxel	9	16.70%
	No chemotherapy given	33	61.10%
Age in years, median (range)		38.5 (13-77)	
Duration of symptoms in months, median (range)		5 (1-22)	

**Figure 2:**
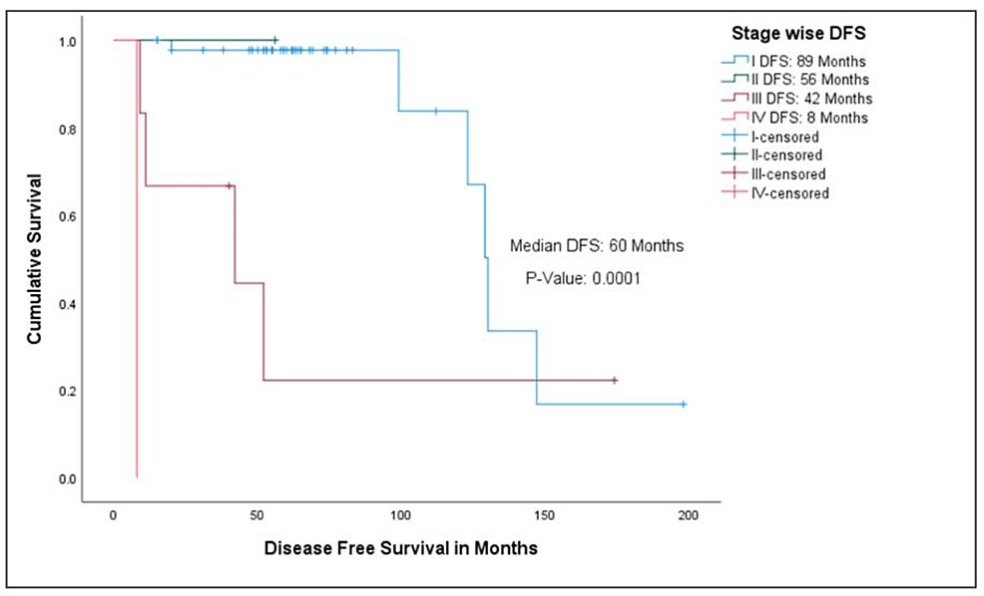
Kaplan-Meier curve showing disease-free survival for FIGO stage I to IV DFS - disease-free survival

**Figure 3:**
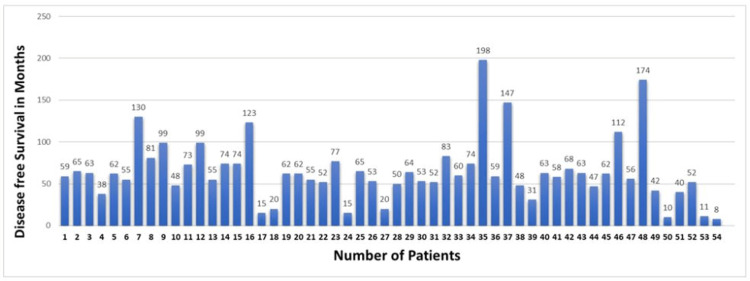
Bar chart showing disease-free survival for all the patients included in this study Patients number 1 to 46 are stage I, patient number 47 is stage II, patients number 48 to 53 are stage III, and patient number 54 is stage IV.

Overall median disease-free survival was 60 months for all the stage groups, while it was 89, 56, 42, and eight months from stage I to IV, respectively (Figures 2, 3).

Eleven patients relapsed with abdominal pain as the most common presenting symptom in 64% (n=7), followed by abdominal distension in 27% (n=3). The rest (9%, n=1) were asymptomatic. Out of these eleven patients, six patients had residual disease in their post-operative scans. Imaging revealed an abdominal mass in 45% (n=5), extensive peritoneal disease in 36% (n=4), and a left adnexal mass in 18% (n=2) of the patients. Second-look debulking surgery was performed in 36.3% (n=4) and completion surgery in 18% (n=2) of the patients (who previously had a simple total hysterectomy). Adjuvant chemotherapy was given to 73% (n=8), one patient refused further treatment, and two patients were not fit for any treatment because of poor performance status (Table 2).

**Table 2:** Patient and tumor characteristics after first treatment relapse in comparison with the outcome (alive/dead)

Patient and tumor characteristics after first relapse		Number of patients	Percentage	Status	
				Alive	Dead
Eastern Cooperative Oncology Group performance status (ECOG)	0	9	81.8%	4	5
	1	1	9.1%	1	0
	3	1	9.1%	0	1
Parity	Nulliparous	1	9.1%	0	1
	Uniparous	0	0.0%	0	0
	Multiparous	10	90.9%	5	5
Menstrual status	Postmenopausal	8	72.7%	3	5
	Premenopausal	3	27.3%	2	1
Type of granulosa cell tumor	Adult	11	100.0%	5	6
Residual disease after first-line treatment	No	6	54.5%	3	3
	Yes	5	45.5%	2	3
Image findings at first relapse	Abdominal mass	5	45.5%	1	4
	Extensive peritoneal disease	4	36.4%	2	2
	Left adnexal mass	2	18.2%	2	0
Treatment offered after first relapse	No	1	9.1%	0	1
	Yes	10	90.9%	5	5
Chemotherapy given after first relapse	Bleomycin/ etoposide/ cisplatin	2	18.2%	1	1
	Carboplatin/ paclitaxel	5	45.5%	3	2
	Epirubicin/ cyclophosphamide	1	9.1%	0	1
	No	2	18.2%	0	2
	Refused	1	9.1%	1	0
Surgery after first relapse	Completion surgery	2	18.2%	1	1
	Debulking	4	36.4%	3	1
	No	5	45.5%	1	4
Baseline FIGO stage	IA	5	45.5%	3	2
	IC	1	9.1%	1	0
	IIIC	4	36.4%	0	4
	IVB	1	9.1%	1	0
Status	Alive	5	45.5%		
	Dead	6	54.5%		

Major factors that contributed towards decreased progression-free survival were post-menopausal status, advanced disease at presentation, capsular rupture, presence of ascites, omental involvement, peritoneal spread, and residual disease after surgical resection (Table 3).

**Table 3:** Patient and tumor characteristics that contributed to relapse after the first treatment

Patient Characteristics		Number of patients relapse after first treatment			
		Total	No	Yes	p-value
Eastern Cooperative Oncology Group performance status (ECOG)	0	47	38 (81%)	9 (19%)	0.135
	1	6	5 (83.3%)	1 (16.7%)	
	3	1	0 (0%)	1 (100%)	
Menstrual status	Postmenopausal	16	8 (50%)	8 (50%)	0.001
	Premenopausal	38	35 (92.1%)	3 (14.8%)	
Parity	Nulliparous	10	9 (90%)	1 (10%)	0.22
	Uniparous	6	6 (100%)	0 (0%)	
	Multiparous	38	28 (73.7%)	10 (26.3%)	
Duration	Less than 3 months	16	12 (75%)	4 (25%)	0.85
	3-6 months	21	17 (81%)	4 (19%)	
	More than 6 months	17	14 (82.4%)	3 (17.6%)	
Laterality	Bilateral	11	8 (72.7%)	3 (27.3%)	0.76
	Left	24	20 (83.3%)	4 (16.7%)	
	Right	19	15 (79%)	4 (21%)	
Type of surgery	Bilateral salpingo-oophorectomy	1	0 (0%)	1 (100%)	0.04
	Completion surgery	26	23 (88.5%)	3 (11.5%)	
	Debulking surgery	2	0 (0%)	2 (100%)	
	Fertility sparing surgery	6	5 (83.3%)	1 (16.7%)	
	Left salpingo-oophorectomy	6	4 (66.7%)	2 (33.3%)	
	Right salpingo-oophorectomy	3	3 (100%)	0 (0%)	
	Total abdominal hysterectomy + bilateral salpingo-oophorectomy	9	7 (78%)	2 (22%)	
	Total abdominal hysterectomy + left salpingo-oophorectomy	1	1 (100%)	0 (0%)	
Type of granulosa cell tumor	Adult	46	35 (76.1%)	11 (23.9%)	0.12
	Juvenile	8	8 (100%)	0 (0%)	
Residual disease	No	48	42 (87.5)	6 (12.5%)	0.001
	Yes	6	1 (16.7%)	5 (83.5%)	
Tumor size	<10cm	27	22 (81.5%)	5 (18.5%)	0.73
	>10cm	27	21 (77.8%)	6 (22.2%)	
FIGO stage	I	46	40 (87%)	6 (13%)	0.003
	II	1	1 (100%)	0 (0%)	
	III	6	2 (33%)	4 (67%)	
	IV	1	0 (0%)	1 (100%)	
Capsule rupture at surgery	Intact	35	31 (88.6%)	4 (11.4%)	0.008
	Ruptured	19	12 (63.2%)	7 (36.8%)	
Peritoneal spread	Free of tumor	47	41 (87.2%)	6 (12.8%)	0.001
	Positive	7	2 (28.6%)	5 (71.4%)	
Omental involvement	Free of tumor	47	41 (87.2%)	6 (12.8%)	0.001
	Positive	7	2 (28.6%)	5 (71.4%)	
Ascites	No	50	42 (84%)	8 (16%)	0.005
	Yes	4	1 (25%)	3 (75%)	
Chemotherapy regimen	Bleomycin/ etoposide/ cisplatin	8	4 (50%)	4 (50%)	0.2
	Carboplatin/ cyclophosphamide	1	1 (100%)	0 (0%)	
	Carboplatin/ etoposide	3	3 (100%)	0 (0%)	
	Carboplatin/ paclitaxel	9	8 (89%)	1 (11%)	
	Nil	33	27 (82%)	6 (18%)	

**Figure 4:**
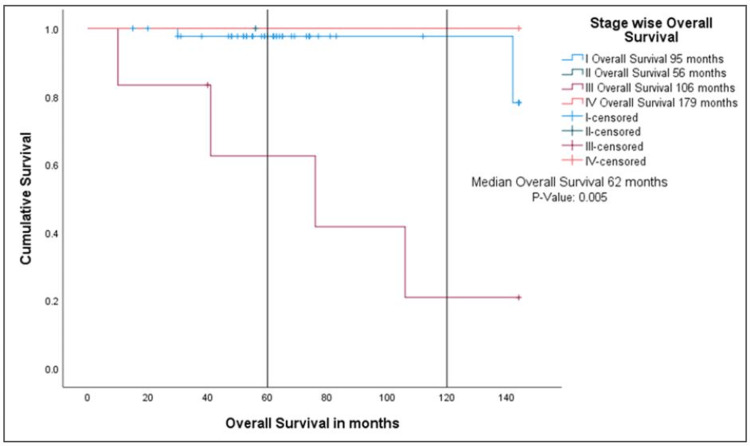
Kaplan-Meier curve showing overall survival for FIGO stages I to IV

**Figure 5:**
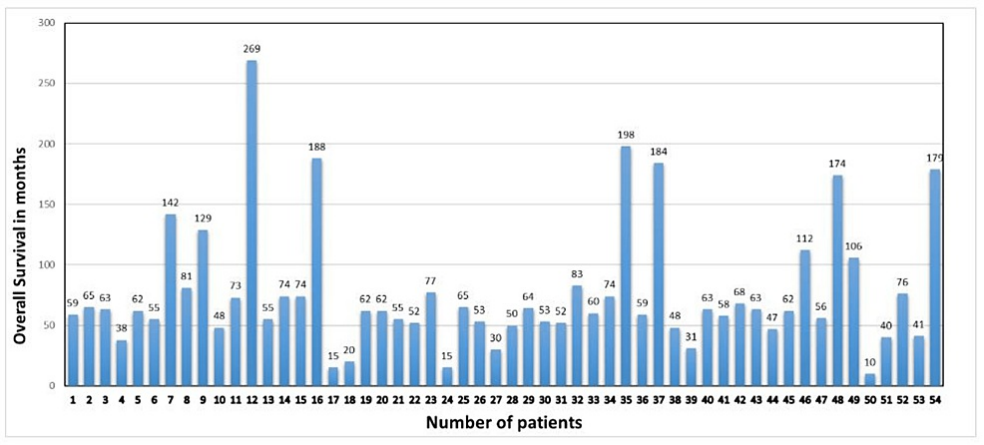
Bar Chart showing disease-free survival for all the patients included in this study Patients number 1 to 46 are stage I, patient number 47 is stage II, patients number 48 to 53 are stage III, and patient number 54 is stage IV.

Out of these 11 patients, three patients went into remission, two patients still have active disease, and six died. Out of these six dead patients, four patients had advanced disease at presentation, the remaining two patients had stage IA, and both had multiple relapses before death suggesting better survival outcomes with early-stage disease. Overall survival was 62 months in all stage groups, while it was 95, 56, 106, and 179 months for stages I to IV, respectively (Figures 4, 5).

## Discussion

Literature suggests that granulosa cell tumors are common in post-menopausal women and are usually diagnosed at an early stage resulting in overall better survival outcomes [8]. In this study, the median age was 38.5 years (range: 13-77 years), and around 30% of the patients were post-menopausal, which contrasts with the data available. We also noted that pre-menopausal patients had a better disease-free survival outcome (p-value: 0.001, HR 0.11, 95% CI: 0.03- 0.42).

Sehouli et al. [8] reported the common clinical manifestations are abdominal pain/distention, a pelvic mass, and vaginal bleeding. While our study found that dysfunctional uterine bleeding (n=22, 40.7%) and abdominal pain (n=22, 40.7%) were the most common presenting symptoms, while abdominal distention (n=8, 14.8%) was the second most common presentation.

Park et al. suggested that none of the patients who underwent complete surgical staging had a relapse, and 100% optimal debulking was achieved even in advanced disease; recurrences were only seen in patients with post-operative residual disease [9]. Patients with granulosa cell tumors with an early-stage presentation where optimal debulking was achieved with no residual disease in post-operative imaging resulted in better overall survival and disease-free survival outcomes [10]. Most of our patients underwent complete debulking surgery as per ovarian protocol (n=26, 48%); the rest of the patients (n=28, 52%) underwent simple TAH+BSO, debulking surgery, unilateral salpingo-oophorectomy, and fertility-sparing surgery. Post-operative imaging suggested that 89% (n=48) patients had no residual disease, out of which only 12.5% (n=6) of the patients relapsed, suggesting better disease-free survival outcomes in patients with no residual disease post-surgery (p-value: 0.001, HR 3.2, 95% CI: 1.78-5.9). As it is established in the literature, patients who presented at an early stage had better disease-free survival outcomes as compared to those who presented at an advanced stage (p-value 0.003).

Markman et al. established the role of progression-free survival as a principal factor in predicting chemotherapy sensitivity and prognosis in recurrent epithelial ovarian cancer; however, this has not been clarified in recurrent granulosa cell tumors owing to its rarity, early-stage presentation, late relapses, and relatively poor sensitivity to chemotherapy [11-12]. In this study, chemotherapy was given to only 39% (n=21), and regimens used were bleomycin/ etoposide/ cisplatin (BEP), carboplatin/ cyclophosphamide, carboplatin/ etoposide, and carboplatin/ paclitaxel, and no significant difference was noted in terms of disease-free survival (p-value: 0.2, HR 0.74, 95% CI: 0.41-1.34). Chan et al. reported that at diagnosis, a tumor size of more than 10 cm confers a poor prognosis [13]. This was also supported by Miller et al. [14], who found that the recurrence was more significant with a larger tumor size (13.5 cm vs. 10 cm; p=0.029). However, this study did not show any statistical difference in disease-free survival with a tumor size of more than 10 cm (p-value: 0.73, HR 0.91, 95% CI: 0.50-1.65).

Our study also suggested that capsule rupture at surgery (p-value: 0.008, HR 2.2, 95% CI: 1.2-4.2), peritoneal spread (p-value: 0.001, HR 3.1, 95% CI: 1.70-5.7), omental involvement (p-value: 0.001, HR 3.1, 95% CI: 1.70-5.7) and presence of ascites (p-value: 0.004, HR 0.35, 95% CI: 0.17-0.71) also confer poor disease-free survival outcomes.

Residual disease post-surgery is another factor deciding disease-free survival in patients with granulosa cell tumors, as reported by Al-Badawi et al. [15]. Our study found out that six patients had residual disease post-surgery, and five of them relapsed later with a statistically significant p-value (p-value: 0.001, HR 3.2, 95% CI: 1.78-5.9)

Patients with recurrent granulosa cell tumors show better survival outcomes if complete debulking is achieved with or without adjuvant chemotherapy [16]. There are no established guidelines for post-operative treatment; multiple approaches like chemotherapy, radiotherapy, and hormonal therapies have been proposed [17-19]. A decision to offer post-operative chemotherapy is based on the tumor stage and the presence of residual disease. Chemotherapy can be offered to patients with advanced-stage disease with or without suboptimal cytoreduction. Hormone therapy also has been tried for refractory granulosa cell tumors with variable results [20].

In our study, 11 patients relapsed after primary treatment, out of which two patients had a complete resection, and four had debulking surgery. Second-line chemotherapy was given to eight patients, and five patients are alive with active surveillance follow-up.

We also had two patients with frequent relapses; both had multiple relapses, with the one patient being diagnosed with hepatocellular carcinoma as a sequela of cirrhosis related to hepatitis C infection in the past, for which she was offered radiofrequency ablation with no residual disease on follow-up scans. The second patient had cytoreductive surgeries after the first and second relapses with chemotherapy (carboplatin/ paclitaxel). Both patients are currently on hormonal treatment with stable disease on follow-up imaging with median overall survival exceeding fifteen years.

Owing to its late relapses, continuous long-term follow-up is needed; it would be an easily forgotten tumor if not followed up regularly, and recurrences have been reported even after 22 years [21]. Most recurrences are abdominal and intraperitoneal; surveillance with abdominal and pelvic imaging is necessary. Serum Beta inhibin levels can also be used as a tumor marker during follow-up for recurrence detection [22].

Limitations of this study are its retrospective design and small patient population. A retrospective analysis of 54 patients is too small to draw definitive conclusions about this rare entity. This might form the basis for further prospective trials and large-scale retrospective studies outlining baseline characteristics, prognostic factors, and outcomes of this disease, preferably in a multi-center collaboration.

## Conclusions

This study shows that post-menopausal patients, more advanced disease at presentation, capsular rupture, presence of ascites, omental involvement, peritoneal spread, and residual disease after surgical resection were the main contributing factors towards poorer outcomes affecting disease-free survival. Optimal debulking surgery in recurrent granulosa cell tumors adds to survival outcomes. Furthermore, long-term follow-up is suggested because of its late relapses and multiple recurrences.
